# ICP curve morphology and intracranial flow-volume changes: a simultaneous ICP and cine phase contrast MRI study in humans

**DOI:** 10.1007/s00701-017-3435-2

**Published:** 2017-12-22

**Authors:** Mårten Unnerbäck, Johnny T. Ottesen, Peter Reinstrup

**Affiliations:** 1 Department of Clinical Sciences Lund, Intensive Care and Perioperative Medicine, Lund University, Skane University Hospital, Malmö, Sweden; 2 Department of Science and Environment, Roskilde University, Roskilde, Denmark; 3 Department of Clinical Sciences Lund, Department of Neurosurgery, Lund University, Skane University Hospital, Lund, Sweden

**Keywords:** Intracranial pressure, Cerebral blood flow, Magnetic resonance imaging, Intracranial compliance

## Abstract

**Background:**

The intracranial pressure (ICP) curve with its different peaks has been extensively studied, but the exact physiological mechanisms behind its morphology are still not fully understood. Both intracranial volume change (ΔICV) and transmission of the arterial blood pressure have been proposed to shape the ICP curve. This study tested the hypothesis that the ICP curve correlates to intracranial volume changes.

**Methods:**

Cine phase contrast magnetic resonance imaging (MRI) examinations were performed in neuro-intensive care patients with simultaneous ICP monitoring. The MRI was set to examine cerebral arterial inflow and venous cerebral outflow as well as flow of cerebrospinal fluid over the foramen magnum. The difference in total flow into and out from the cranial cavity (Flow_tot_) over time provides the ΔICV. The ICP curve was compared to the Flow_tot_ and the ΔICV. Correlations were calculated through linear and logarithmic regression. Student’s *t* test was used to test the null hypothesis between paired samples.

**Results:**

Excluding the initial ICP wave, P1, the mean *R* ^2^ for the correlation between the ΔICV and the ICP was 0.75 for the exponential expression, which had a higher correlation than the linear (*p* = 0.005). The first ICP peaks correlated to the initial peaks of Flow_tot_ with a mean *R* ^2^ = 0.88.

**Conclusion:**

The first part, or the P1, of the ICP curve seems to be created by the first rapid net inflow seen in Flow_tot_ while the rest of the ICP curve seem to correlate to the ΔICV.

## Introduction

Lundberg [22] introduced the continuous monitoring of the intracranial pressure (ICP) in 1960 and this progress is today one of the most important aspects within neuro-intensive care [12]. The ICP curve follows the cardiac cycle and usually displays a variable amount of peaks. The peaks were initially interpreted as secondary to heart valves opening and closing during the cardiac cycle. Usually there are four peaks and Gega et al. proposed naming them P1, P2, P3, and so on [11, 16].

Early investigators pointed toward the possibility that the ICP curve is mainly shaped by the arterial blood pressure wave traveling through the arteries and then being transferred into the intracranial cavity [9, 15]. To support this theory, it was early shown that extirpation of the plexus arachnoideus dampened the ICP curve, a finding that was interpreted as the result of less pressure transferring from the arteries to the intracerebral ventricles [9]. Other investigators concluded that it is the venous pressure that shapes the ICP curve [18]. Experiments in dogs lead to the conclusion that both the arterial and venous pressure waves affected the ICP curve [2].

Since the cranial cavity is a closed compartment, the ICP curve could be a product of intracranial volume (ICV) changes [3]. Previous investigations with phase contrast MRI found changes in ICV over the cardiac cycle, by measuring the arterial inflow; venous outflow, and cerebrospinal fluid (CSF) flow over the foramen magnum [3, 5, 8]. Initially, the arterial inflow supersedes the venous and CSF outflow generating an increase in ICV. However, the Monroe-Kellie doctrine states that the intracranial volume cannot change due to the incompressibility of the intracranial content, so if the measured change in ICV volume due to blood and CSF flow is true, there must be another change in intracranial volume to compensate for this. Through dynamic MRI technology, it has been shown that the intracranial parenchyma moves pulsatile in a rostral-caudal direction during the cardiac cycle [17]. Since the ICV changes are very small, it is feasible that this motion compensates the volume change, dampening ICP changes. This change in ICV may however not solely explain the morphology of the ICP curve since the change in ICV is slower in onset, peaks much later in the cardiac cycle than the ICP curve, and lacks the characteristic peaks [3].

It has been shown that the intracranial flow initially rises due to the arterial inflow and later on falls as the CSF and venous outflow compensates increasingly for this [3]. This generates a minimal but rapidly increasing ICV change (ΔICV). This rapid flow could produce a substantial change in ICP since the only compensatory mechanism would be a slightly temporally delayed movement of the intracranial parenchyma caudally driven by the rise in ICP [17].

Although much work has been done regarding the intracranial compliance and compensatory mechanisms [20, 21, 27–29], less has been reported about the physiological mechanisms underlying the ICP changes over a cardiac cycle [3, 13, 31, 32].

We therefore wanted to investigate the flow in and out of the cranial cavity during a pulse cycle, the resulting ΔICV, and correlate it to simultaneous measured intraventricular ICP recordings in neuro-intensive care patients.

## Materials and methods

### Inclusion criteria

Ethical approval for the study was granted by the Regional Ethical Review Board at Lund University. (2014/403) Patients admitted to the neuro-intensive care unit at the Skånes University Hospital during the period 2014–2017, monitored with an intra ventricular ICP catheter and clinical requirement of a MRI investigation were included. Patients with a non-intact cranial cavity, except for the insertion hole of the ventricular catheter, were excluded.

### MRI examinations

MRI examinations were in all cases performed using a Philips Intera 1.5T. Slices, placed just under the foramen magnum perpendicular to the vessel, were 6 mm thick and a 256 × 128 matrix was used. The velocity encoding value was set to 90 cm/s for blood flow and 8 cm/s for CSF during the phase contrast MRI examination. The flip angel was set to 15°. TR was 26 ms. Each cardiac cycle was sampled at 30 to 35 time points and the total examination time was 2 min. The arterial and venous blood flow was measured immediately before the CSF measurements, during a physiological stable time period.

All MRI examinations were analyzed using the freely available software SEGMENT v 2.0 R5432 [19]. One examiner, blinded to the ICP curves, performed the analysis of the MRI examinations. The region of interests of the internal carotid arteries, vertebral arteries, internal jugular veins, and the cerebrospinal canal were outlined manually and the flow were acquired in the phase contrast images pixel by pixel with a temporal resolution of 30 to 35 slices per cardiac cycle. The total flow in the carotid and vertebral arteries was summarized yielding the arterial cranial inflow. The flow in the internal jugular veins was summarized for estimation of the cranial venous outflow.

Venous outflow from the intracranial compartment may take different paths out of the cranial cavity and the proportion flowing through the internal jugular veins varies between individuals [14]. In order to determine the total venous outflow, the jugular venous outflow was multiplied by a factor to equal the arterial inflow over a cardiac cycle [3].

The flow during the cardiac cycle (Flow_tot_) was calculated at each time point by subtracting venous and CSF outflow from arterial inflow. The change of the intracranial volume over time was calculated by multiplying Flow_tot_ with time, resulting in a volume change and then summarizing these volume changes over time, described in the equation:

$$ \Delta ICV\left(\tau \right)={\int}_{\tau_0}^{\tau}\left( CBFa(t)- CBF\nu (t)- CSFF(t)\right) dt $$

where ΔICV is the change in intracranial volume, CBFa is the arterial cerebral blood flow, CBFv is the venous cerebral blood flow, and CSFF is the reverberating flow of CSF through the foramen magnum.

### ICP measurements

The ICP had in all patients been measured with a 8-F tunneled intraventricular catheter (HanniKath, Smiths Medical Deutschland GmbH), inserted through a burr hole cranially. The catheter had been connected to a CSF drainage set with a microtransducer (HanniSet, Smiths Medical Deutschland GmbH). The transducer was zeroed against atmospheric pressure at the uppermost point of the cranium. The signal from the ICP pressure transducer was taken out of the MR-investigation room through a radiofrequency filter at the penetration panel in the shielding enclosure into the MR-control room. In the control room, the values were digitally registered with a Philips Intellivue MP70 STAD at a sampling rate of 125 Hz and stored in a database. The ICP curves registered during the MRI examinations were identified and analyzed manually. All ICP curves during one respiratory cycle were summarized and a mean ICP curve calculated. This ICP curve was compared to the concurrent ΔICV derived from the MRI examinations using the lowest ICP and the lowest ΔICV as starting point.

### Statistical analysis

All statistical analysis was performed using IBM SPSS Statistics for Windows, Version 22.0. (IBM Corp, USA) All values are given as mean and standard deviation. Linear regression was used to establish goodness of fit and to establish the optimal expression, either linear or logarithmic. Student’s *t* test was used to test the null hypothesis between paired samples.

All values are presented as Mean ± SD unless stated otherwise.

## Results

Mean age of the included 10 patients was 48.6 ± 11.4 years and 9 were male. The mean ICP value of the examinations was 16 ± 10 mmHg. Maximum ΔICV (ΔICV_max_) was 0.68 ± 0.33 ml.

**Fig. 1:**
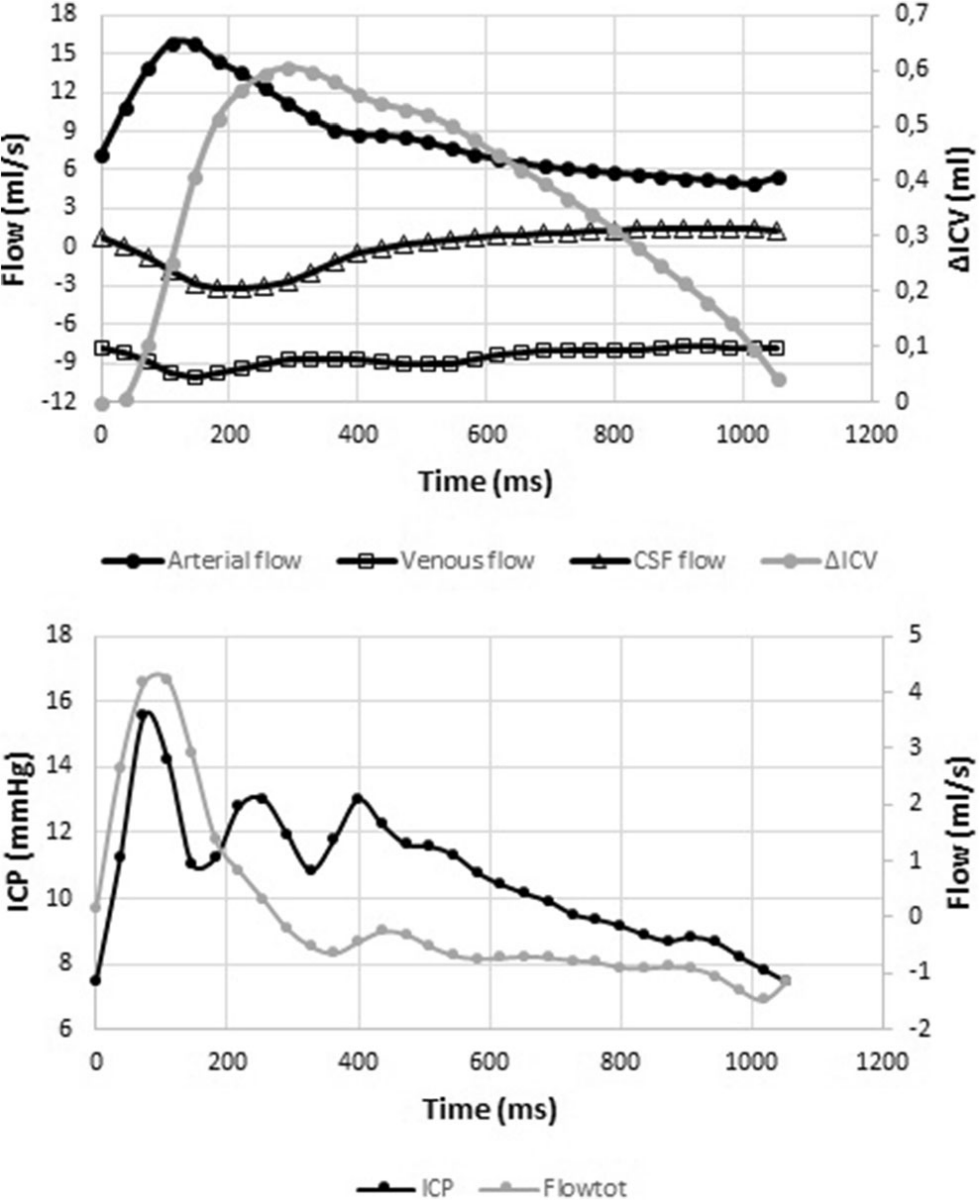
The upper graph shows arterial flow, venous flow, CSF flow, and the resulting intracranial volume change (ΔICV) over time. The lower graph shows the total flow (Flow_tot_) and the ICP curve. All data are from the same individual and plotted over the same timeline in the two graphs

Total flow varied over time, with typically two or more peaks over a cardiac cycle. (Fig. 1).

Using linear regression, there was a correlation between ICP and ΔICV, *R* ^2^ = 0.41 ± 0.14. The correlation was strengthened using a logarithmic regression line, *R* ^2^ = 0.47 ± 0.13. The correlation between ICP and ΔICV using logarithmic regression was stronger compared to using a linear regression (*p* = 0.005).

**Fig. 2:**
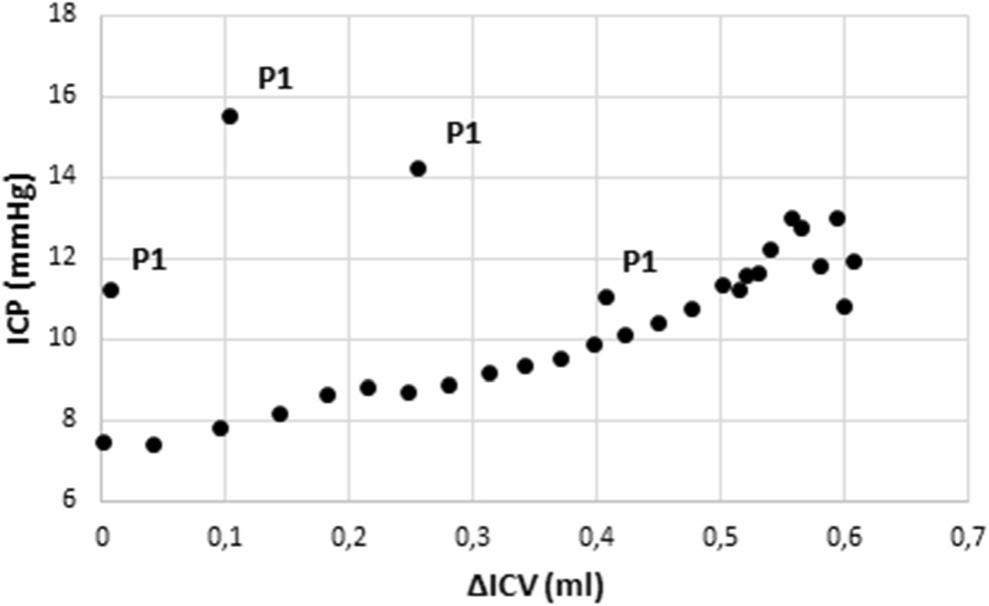
Regression plot of intracranial pressure (ICP) against intracranial volume change (ΔICV) in one individual. The points corresponding to the initial peak (P1) are apparent as outliers and marked “P1”

The values representing P1 can be clearly identified as outliers when ICP is plotted against ΔICV (Fig. 2). In all examinations, but one, the correlation between ΔICV and ICP was reinforced when P1 was excluded. Using a logarithmic expression excluding P1 resulted in a mean *R* ^2^ of 0.75 ± 0.15, statistically significant compared to not excluding P1 (*p* = 0.001).

**Fig. 3:**
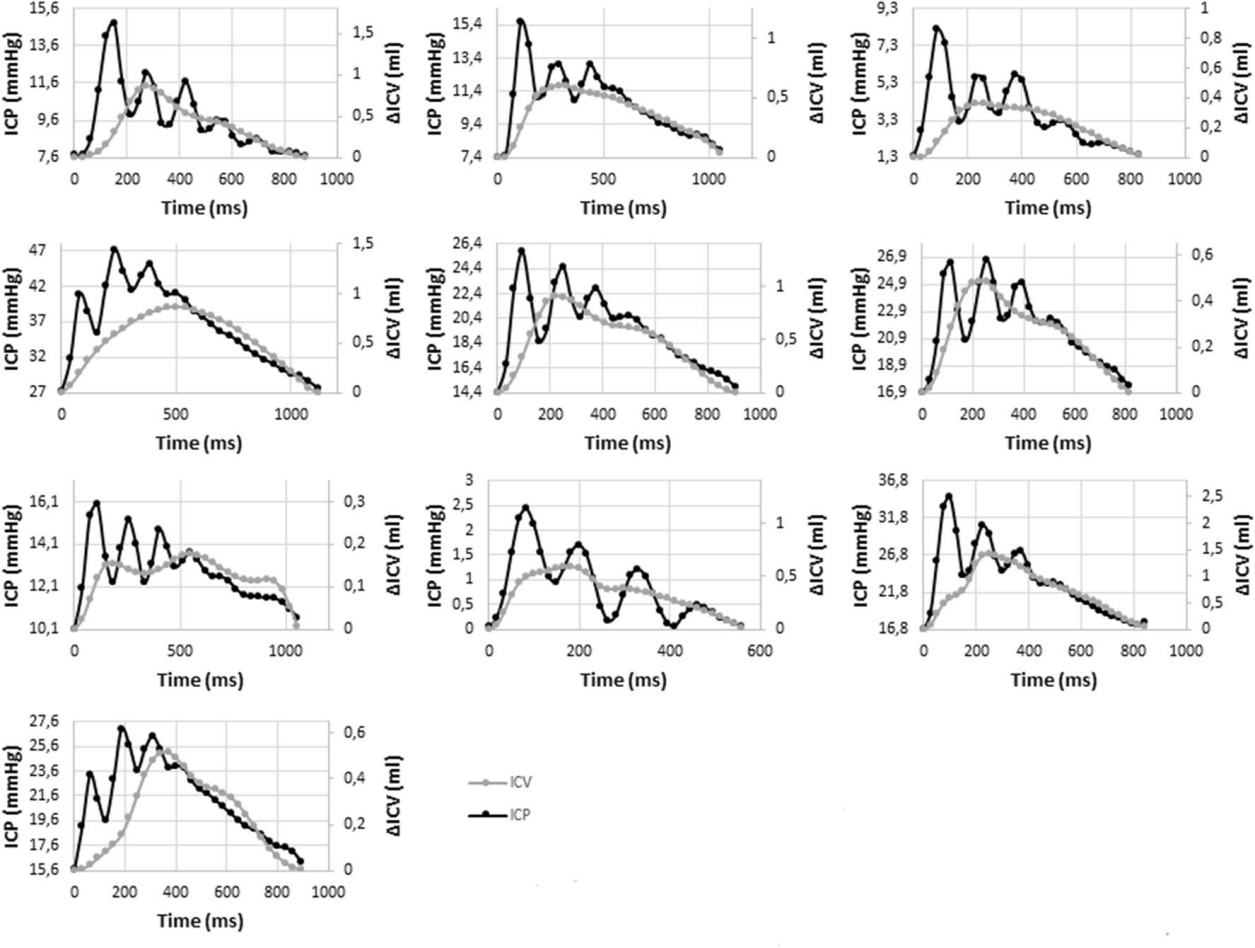
Intracranial pressure (ICP) and ΔICV plotted against time for all examinations

Plotting the ΔICV against ICP resulted in a graph where the ΔICV curve follows in close proximity of the ICP curve, except for the initial P1peaks (Fig. 3).

Comparing the total flow values during the initial peak of the flow curve with the ICP values of the P1 resulted in a correlation with a mean *R* ^2^ of 0.88 (± 0.10). The mean temporal delay between the first flow peak and P1 was 22 ± 28 ms.

## Discussion

The ICV includes three volumes that can change rapidly during a pulse stroke, the cerebral blood volume (CBV), which can be divided in arterial cerebral blood volume (aCBV) and venous cerebral blood volume (vCBV), as well as the cerebrospinal fluid (CSF). We hypothesized that the ICP curve morphology could be a result of the ICV change over time as well as an effect of the rapid inflow into the intracranial space. The data from this study shows a strong exponential correlation between the ICP changes and ΔICV. To our knowledge, this link between concomitant ICV and ICP changes over the cardiac cycle has been proposed, but not fully demonstrated [3, 7, 30]. In a transcranial Doppler (TCD) study, Carrera et al. showed a relation between ΔCBV and ICP [13] when assuming a constant venous outflow based on previous research [1]. However, several studies have demonstrated that the venous outflow from the cranial cavity is pulsatile [3, 4, 25] and Carrera et al. do not compensate for CSF fluctuations, as there was no measurement of CSF flow from the cranial cavity through the foramen magnum secondary to the change in CBV. Consequently, it could be argued that they demonstrated a relation between changes in aCBV and changes in ICP [30].

The ICP curve should more correctly correlate to the ΔICV than the ΔCBV. Since venous outflow from the cranial cavity increases dependent on the arterial inflow into the cranial cavity and the CSF outflow begins as the CBV increase [8, 26], the ΔICV curve peaks later as compared to the method using a constant venous outflow and not taking CSF flows into account. Carrera et al. concluded that the initial part of the ICP curve correlated to the arterial blood pressures (ABP) systolic peak and the subsequent part correlated to ΔCBV [13] or more correctly, as earlier described, to the ΔaCBV. Our findings support a multifactorial origin of the ICP curve morphology as the ΔICV correlated to the ICP in the later part of the curve, but with no correlation to the initial ICP peak.

The exponential relation between ICP and ICV is well known from previous studies; it has been described by the following equation [7, 21, 24, 27]:

$$ \mathrm{ICP}={P}_1\times {e}^{\mathrm{E}\times \mathrm{ICV}} $$

where *P*_1_ represents a pressure coefficient, *E* represents the elastance coefficient, and ICV equals the intracranial volume. This relationship has previously been used for studying the relationship between ICV and ICP over the cardiac cycle in humans [13, 32]. Our data supports the hypothesis that the exponential nature of this relationship is true over the small volume changes taking place during the cardiac cycle, since the strongest relationship was found between ΔICV and the ICP curve when described as an exponential function.

There is some evidence that P2 and P3 are caused by changes in aCBV and therefore dependent on the elastance of the craniospinal system [13]. Examining the graph plotting ΔICV against ICP (Fig. 3), it seems that patients in our study with a P1/P2 amplitude ratio above 0.9 had a slower rise in ΔICV, which consequently peaked later during the cardiac cycle. This slow change was attributed to high venous and CSF outflow rates and not to low arterial inflow. The two individuals with elevated ICP levels, but without an elevated P2, did not have this slow change in ΔICV. Although our study is too small to find a statistically significant difference, we find this interesting.

It has been suggested that the initial sharp peak (P1) is a result of the transfer of the arterial blood pressure into the cranial cavity [9, 11, 15]. Since the ICP curve with its multiple peaks does not fully resemble the arterial pressure curve, there must be other factors affecting it. Rapid changes of intracranial flow could also produce a sharp peak. As the content of the cranial cavity is virtually incompressible, a small increase in ICV would lead to a large rise in ICP, an effect that could be dampened by other less well-examined factors referred to as brain elastance. If there is an inertia in this dampening system, the inflow could cause a small but rapid change in ICV and thereby ICP, since the compensatory mechanisms are put in effect with a slight delay. As the ΔICV rate of change, i.e., Flow_tot_, decelerates due to the venous and CSF outflow, the dampening become sufficient and the ICP falls rapidly resulting in a peak. The delay in the response to the raise in ICP could cause resonance in the system, which as a result could lead to the oscillations observed around the increased ICP due to the ΔICV increase. The subsequent peaks observed could thereby be the result of the interaction between arterial inflow, venous outflow, CSF flow over the foramen magnum, and the intracranial elastance. These changes are probably minor, which would explain why we couldn’t measure them fully with the present method.

It is possible that a stronger correlation could have been found if we had chosen a regression line other than logarithmic or linear. This was considered, but we concluded that the logarithmic or linear lines served the purpose of this study best.

In this study, we did not have the possibility to record the ABP curve simultaneously. Since the ICP curve seems to be a compound of several different factors, it would be of interest to include this in future studies.

The technique for measuring the arterial, venous, and CSF flows, using MRI, has been used previously, but not in conjunction with simultaneous ICP recording. The MRI flow measurements have been demonstrated to have methodological errors of max 10%. [10, 23] It should however be stressed that the volumes measured are small and minor errors could affect the data, probably in a systematic way. The changes in ΔICV measured are within what have been predicted to be in the physiological range [6]. Our conclusion is that the shape of the measured flow curve could be regarded as consistent with the shape of the actual flow curve.

The study has a limited sample size and further studies with this methodology could provide more robust and precise answers.

## Conclusion

Our data suggests that the change in the ICP curve during a pulse stroke is composed from different cerebrovascular flow parameters. The first peak of the ICP curve correlated well to the initial arterial blood flow into the cranial cavity, opening for the possibility that the primary part of the ICP curve is, in some part, created by this flow. The latter parts of the ICP curve correlated to the CBV changes over the cardiac cycle, a correlation that seems to be of an exponential nature.

## Abbreviations

aCBV: Arterial cerebral blood volume
CBFa: Arterial cerebral blood flow
CBFv: Venous cerebral blood flow
CBV: Cerebral blood volume
CSF: Cerebrospinal fluid
CSFF: Cerebrospinal fluid flow
*E*: Elastance coefficient
Flow_tot_: Total flow into and out from the cranial cavity
ICP: Intracranial pressure
ICP(ΔICV): ICP as a function of ΔICV
ICV: Intracranial volume
MRI: Magnet resonance imaging
*P*_1_: pressure coefficient
SD: Standard deviation
TR: Repetition time
vCBV: Venous cerebral blood volume
ΔICV: Intracranial volume change
ΔICV_max_: Maximal intracranial volume change
